# Seasonal influenza among children diagnosed by their guardians: a small pilot study in Japan

**DOI:** 10.1017/S1463423618000245

**Published:** 2018-04-15

**Authors:** Hiroki Maita, Tadashi Kobayashi, Hiroshi Osawa, Hiroyuki Kato

**Affiliations:** 1 Development of Community Healthcare, Hirosaki University Graduate School of Medicine, Hirosaki-shi, Japan; 2 General Medicine, Hirosaki University Graduate School of Medicine, Hirosaki-shi, Japan; 3 Department of General Medicine, Hirosaki University School of Medicine & Hospital, Hirosaki-shi, Japan

**Keywords:** clinical symptoms, explanatory models, parents, quantitative, rapid influenza diagnostic test (RIDT), self-diagnosis

## Abstract

**Aim:**

We aimed to elucidate the accuracy and optimal cut-off point of the self-diagnosis of influenza and the associated clinical symptoms of children by their guardians, compared with those of the rapid influenza diagnostic test (RIDT).

**Background:**

Seasonal influenza is a common outpatient problem during the winter season. A paediatric influenza epidemic has socio-economic impacts like temporary school closure, school event cancellations, and unscheduled work absences among parents. Hence, early identification and assessment of influenza to prevent its spread is important from a societal perspective.

**Method:**

We performed a cross-sectional observational study in a rural clinic in Japan every winter season from December 2013 to March 2016. We retrospectively extracted information from the medical records and pre-examination checklists of 24 patients aged <12 years (mean age, 5.4 years; men, 54.2%). The data extracted from the medical records and pre-examination checklist included the baseline characteristics (age, sex and past medical history of influenza), clinical signs and symptoms, diagnosis by guardians (%) and RIDT results.

**Findings:**

The optimal cut-off point of the self-diagnosis of influenza by guardians was 80%, with a sensitivity and specificity of 63.6% (95% confidence interval: 30.8–89.1) and 92.3% (64.0–99.8). At a 50% cut-off point, the sensitivity and specificity were 90.9% (58.7–99.8) and 53.8% (25.1−80.8). The accuracy of feeling severely sick, as estimated by the guardians showed a sensitivity and specificity of 90.9% (58.7–99.8) and 69.2% (38.6–90.9). Our study indicates that the diagnosis of seasonal influenza by guardians to their children would be useful in the establishment of both confirmatory diagnoses when it has high probability above the optimal cut-off point (80%), and exclusion diagnosis when it has low probability (50%). Not feeling severely sick, estimated by the guardians might be a useful indicator for the exclusion of paediatric influenza.

## Introduction

Seasonal influenza is a common outpatient problem during the winter season (Infectious Disease Surveillance Center, 2016). In 2010, among the patients who were less than five years old in Japan, 2.3 million were influenza-associated outpatients (Infectious Disease Surveillance Center, 2010). Moreover, 1.9/1000 influenza-associated hospitalizations occur annually. Meanwhile, in the United States, 1.1/1000 influenza-associated hospitalizations occur annually among patients who were less than five years old (Thompson *et al*., 2004). A paediatric influenza epidemic also has socio-economic impacts, such as temporary school closure, school event cancellations and unscheduled work absences among parents (Uchida *et al*., 2013). Hence, early identification and assessment of influenza to prevent its spread is important from a societal perspective.

Although the self-diagnosis of influenza is important in the control and management of the spread of this disease, influenza in children, especially those who are very young, may be difficult to self-diagnose. In New Zealand, the qualitative accuracy of influenza diagnosis by guardians or proxies, in children aged <18 years (one to four years, 47.2%: five to nine years, 21.4%) was reported during the 2009 influenza pandemic (Jutel *et al*., 2011). However, to the best of our knowledge, the quantitative accuracy of self-diagnosis has not been studied. Therefore, we aimed to elucidate the accuracy and optimal cut-off point of the self-diagnosis of influenza by guardians to their children and clinical symptoms compared with those of the rapid influenza diagnostic test (RIDT).

## Methods

We performed a cross-sectional observational study to elucidate the clinical effectiveness of the self-diagnosis of seasonal influenza by guardians to their children in a rural clinic in Towada-shi, Aomori, Japan (Towadako Clinic), which as a population of about 500 individuals (about 30 children aged <12 years).

### Data collection

The patient data for three influenza seasons from December 2013 to March 2016 were retrospectively extracted from medical records and pre-examination checklists. The following three inclusion criteria were applied: (1) aged <12 years old, (2) suspected to have influenza (eg, presence of upper respiratory tract symptoms or fever) and underwent RIDT, and (3) checklist completion by guardians in per cent figures.

The data extracted from the pre-examination checklists, which were filled out before medical consultation, include the baseline characteristics (past medical history of influenza), clinical symptoms (cough, joint and muscle pain, and history of fever including ‘acute or sudden fever’ and ‘gradual fever’ or ‘absence of fever’), symptom duration starting from the onset, severity of feeling sick compared with a common cold (severe, similar or mild), and self-diagnosis (%). Concurrently, the data extracted from the medical records included the baseline characteristics (age and sex), clinical signs (axillary temperature at the clinic and pulse rate), and RIDT results (QuickNavi-Flu, Denka Seiken Co., Ltd., Japan).

### Statistical analysis

The receiver operating characteristic curve was performed to estimate the optimal cut-off point of the self-diagnosis of influenza by guardians. To that end, its sensitivity and specificity were determined using multiple cut-off points. All statistical analyses were conducted using EZR version 1.32 (Saitama Medical Center, Jichi Medical University, Saitama, Japan), which is a modified version of R Commander, a frequently used software in biostatistics that is designed to add statistical functions (Kanda, 2013).

### Ethics statement

A full ethical approval was granted by the Medical Ethics Committee of Hirosaki University (approval number: 2016-1078). All data were fully anonymized at the time of the data collection, and the committee waived the requirement for informed consents. The participation of patients was obtained through an opt-out methodology.

## Results

**Figure 1:**
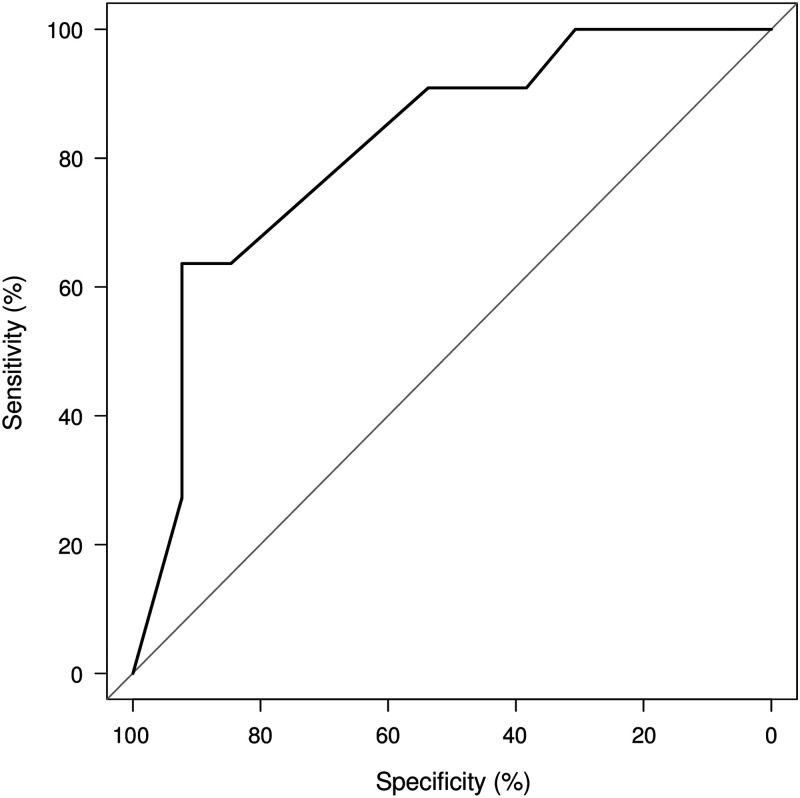
Receiver operating characteristic curves of self-diagnosis by guardians

In our study, the data of 24 patients (mean age, 5.4 years; male, 54.2%) were analysed (Table 1). First, we estimated the accuracy of influenza diagnosis by guardians. The area under the curve (AUC) of the self-diagnosis (%) was 0.82 (95% confidence interval: 0.65–0.99) (Figure 1). The optimal cut-off point was 80%, at which the sensitivity, specificity, positive likelihood ratio (LR+), and negative likelihood ratio (LR–) were 63.6% (30.8–89.1), 92.3% (64.0–99.8), 8.3 (1.2–57.3) and 0.4 (0.2–0.9) (Table 2). At a 50% cut-off point, the sensitivity, specificity, LR+ and LR– were 90.9% (58.7–99.8), 53.8% (25.1–80.8), 2.0 (1.1–3.7) and 0.2 (0.1–1.2) (Table 2). In the subgroup of patients who had been previously infected with influenza (*n*=12), the accuracy of the self-diagnosis by guardians at an 80% cut-off point was estimated to have a sensitivity and specificity of 62.5% (24.5–91.5) and 100% (28.4–100.0).

**Table 1:** Characteristics of the patients (*n*=24)

Mean age [years (SD)]	5.4 (3.2)
Sex [*n* (%)]	
Male	13 (54.2)
Past history of influenza [*n* (%)]	12 (50.0)
Mean axillary temperature on arrival [°C (SD)]	37.7 (0.96)
Pulse rate/min (SD)	108 (26.6)
Cough [*n* (%)]	20 (83.3)
Joint and muscle pain [*n* (%)]	5 (20.8)
History of the fever [*n* (%)]	
Acute or sudden	17 (70.8)
Gradual	7 (29.2)
No fever	0 (0)
Duration from the onset to the rapid influenza testing [h (SD)]	34.9 (22.7)
<12 h [*n* (%)]	4 (16.7)
⩾12 h [*n* (%)]	20 (83.3)
Severity of unpleasant feeling compared with usual cold [*n* (%)]	
Severe	2 (8.3)
Similar	8 (33.3)
Mild	14 (58.3)
Positive for influenza test [*n* (%)]	11 (48.0)
Significant clinical event that required hospitalization [*n* (%)]	0 (0.0)

Items not described in the medical record was counted as none. SD=standard deviation.

**Table 2:** Receiver operating characteristic curve analysis of influenza self-diagnosis by guardians

Cut-off (%)	Sn [% (95% CI)]	Sp [% (95% CI)]
10	100.0 (61.5–100.0)	15.4 (1.9–45.4)
20	100.0 (61.5–100.0)	30.8 (9.1–61.4)
30	90.9 (58.7–99.8)	38.5 (13.9–68.4)
40	90.9 (58.7–99.8)	53.8 (25.1–80.8)
50	90.9 (58.7–99.8)	53.8 (25.1–80.8)
60	63.6 (30.8–89.1)	84.6 (54.6–98.1)
70	63.6 (30.8–89.1)	92.3 (64.0–99.8)
80	63.6 (30.8–89.1)	92.3 (64.0–99.8)
90	27.3 (6.0–61.0)	92.3 (64.0–99.8)

Sn=sensitivity; Sp=specificity; CI=confidence interval.

Second, we validated the accuracy of the axillary temperature and pulse rate. The AUC of the axillary temperature was 0.48 (0.07–0.90), and a statistically significant difference was observed between the self-diagnosis by guardians and axillary temperature (*Z*=–0.74, *P*=0.46). In contrast, the AUC of the pulse rate was 0.63 (0.10–1.00), and a significant difference was not found between the self-diagnosis by guardians and pulse rate (*Z*=–0.35, *P*=0.73). DeLong’s test under a Bonferroni correction was performed twice to compare the correlated AUC, which showed statistically non-significant results. Despite these statistical results, the accuracy of the self-diagnosis by guardians tended to be higher than that of the pulse rate, which did not change the clinical interpretation.

Finally, we examined the accuracy of other clinical signs (Table 3). The presence of joint and muscle pain had an LR+ of 4.73 (0.62–36.3), which was non-significant. In this small-sized pilot study, the sensitivity of fever or cough was 100%. The accuracy of feeling severely sick, as estimated by guardians, had a sensitivity, specificity, LR+, and LR– of 90.9% (58.7–99.8), 69.2% (38.6–90.9), 2.96 (1.28–6.82) and 0.13 (0.02–0.88).

**Table 3:** Accuracy of clinical symptoms of influenza

	Sn [% (95% CI)]	Sp [% (95% CI)]
Cough	100.0 (61.5–100.0)	30.8 (9.1–61.4)
Joint and muscle pain	36.4 (10.9–69.2)	92.3 (64.0–99.8)
History of fever	100.0 (61.5–100.0)	0.0 (0.0–33.9)
Sudden onset fever	87.5 (47.3–99.7)	37.5 (15.2–64.6)
Severity of patient feeling sick^a^		
Severe	90.9 (58.7–99.8)	69.2 (38.6–90.9)
Similar	9.1 (0.20–41.3)	46.2 (19.2–74.9)
Mild	0.0 (0.0–38.5)	84.6 (54.6–98.1)

a As compared with a common cold.

Sn= sensitivity; Sp=specificity; CI=confidence interval.

## Discussion

Our study indicates that the diagnosis of seasonal influenza by guardians to their children would be useful in the establishment of both confirmatory diagnoses when it has high probability above the optimal cut-off point (80%), and exclusion diagnosis when it has low probability (50%). Moreover, the absence of feeling severely sick, as estimated by guardians, might be a useful indicator in the exclusion of paediatric influenza.

### Diagnosis of influenza

Reverse transcription polymerase chain reaction is the gold standard for the diagnoses of influenza. However, this test is usually difficult to carry out, especially in rural clinics in Japan. Therefore, influenza is commonly diagnosed through RIDT (sensitivity, 62.3% [57.9–66.6]; specificity, 98.2% [97.5–98.7]) (Chartrand *et al*., 2012). This test has relatively low sensitivity, especially when used within 12 h from the onset of patient symptoms (Mitamura and Sugaya, 2006). In our study, the average duration from symptom onset to RIDT performance was 34.9 h. More than 80% of patients were examined more than 12 h from symptom onset. Despite the negative RIDT results, patients who were strongly suspected to be infected with influenza were usually re-examined after a sufficient time have passed from the symptom onset. In our study, four patients were re-examined, and one of them had positive result. In the Japanese system where patients have free access to medical institutions, patients with negative RIDT results occasionally consult another physician, especially when they are not satisfied with their results. However, considering that our study was performed in a rural area, the lack of information appears to be very low because patients often have limited access to other medical institutions.

### Diagnosis by guardians and clinical symptoms

**Figure 2:**
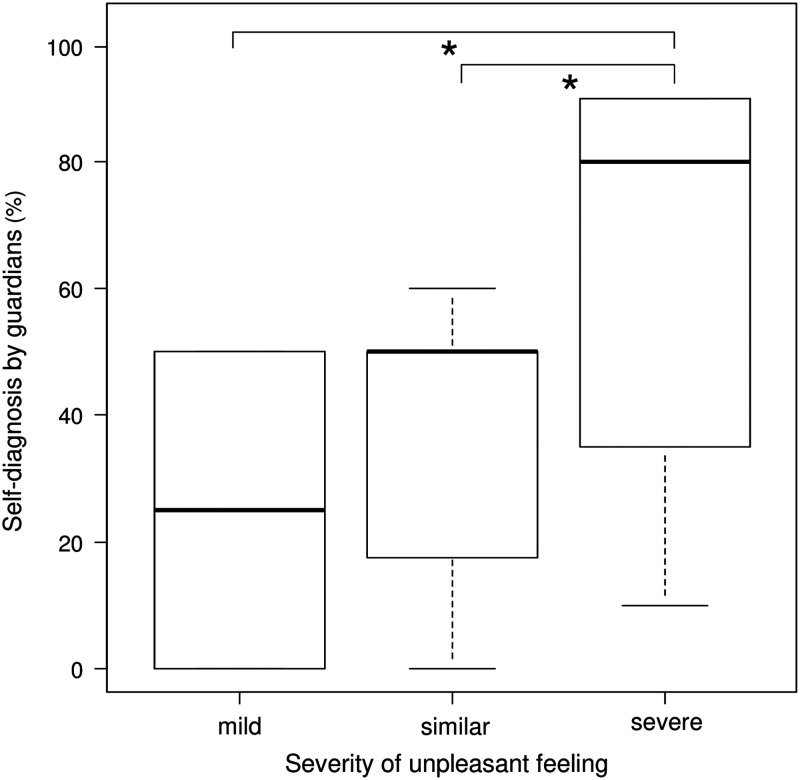
Box plots of self-diagnosis of influenza in children by their guardians for each severity of unpleasant feeling compared with the usual cold. The box plots show the median, upper, and lower quartiles and range. Significant differences (Mann–Whitney *U* test adjusted through the Holm method, *P*<0.05) are indicated by the asterisks.

The AUC of the per cent diagnostic evaluation by guardians was 0.82, which is statistically classified as moderate accuracy. The 80% cut-off point showed high specificity (92.3% [64.0–99.8]). Meanwhile, the 50% cut-off point displayed high sensitivity (90.9% [58.7–99.8]) (Table 2). These results were higher than those of the study conducted by Jutel *et al*. (2011) among children aged <18 years old (sensitivity, 42.4% [30.0–54.9]; specificity, 52.6% [42.1–63.0]) (Jutel *et al*., 2011). The evaluation of severity of feeling sick compared with the usual cold was also useful (Table 3). In fact, as the severity of feeling sick increased, the diagnostic probability also increased (Spearman’s rank correlation *ρ* 0.47, *P*=0.02). To that end, the guardians might estimate the probability of influenza based on the patients’ reported severity of feeling sick (Kruskal–Wallis test, *P*=0.08) (Figure 2). Among the clinical symptoms, the absence of cough or fever was associated with reduced likelihood of influenza, which could be useful in ruling out influenza (Table 3). This finding is similar to that of Jutel and Banister (2013). No other single clinical sign was found to be useful in ruling in or out influenza among the population of our study.

### Diagnostic value of patients’ explanatory models

Self-diagnosis is a part of the patients’ explanatory models (Lang *et al*., 2000), which refer to the notions about an episode of sickness and its treatment that are utilized by all those engaged in the clinical process (Kleinman *et al*., 1978). A better understanding of the parents’ explanatory models can promote an effective communication between physicians and patients (Kai, 1996a; 1996b) and increase the patients’ satisfaction with their medical visits (Lang *et al*., 2000; Robinson and Heritage, 2006). Moreover, the display of empathy by the physicians to the patients’ parents (guardians) can alleviate anxieties (Wasserman *et al*., 1984) and empower them (Kai, 1996a; 1996b). However, the confirmation of the parents’ explanatory models is often believed to be not beneficial in the establishment of a medical diagnosis, except for mental illness. In contrast, the findings of our study indicate that the patients’ explanatory models could result to improved medical diagnosis of physical illness.

### Societal perspectives on diagnosis by guardians

The early detection and prevention of paediatric influenza epidemic is socially important because it has socio-economic impacts, such as temporary school closure, school event cancellations, and unscheduled work absences among parents. An increase of two to three times in the influenza prevalence rate was observed among families with schoolchildren (Woodall *et al*., 1958). In the United States, the total economic burden of influenza epidemics among all ages was reported to be US$ 87.1 billion per year (Klepser, 2014). Meanwhile, school district closures reduced the prevalence rate of acute respiratory illness in a community by 45% and decreased the number of emergency department visits due to influenza (Copeland *et al*., 2013). In Japan, the diagnosis of influenza is important in view of school health policies because schoolchildren with influenza are prohibited from attending school until they can no longer transmit infection to others, based on the School Health and Safety Act. We hope that our study will contribute to the avoidance of an influenza-related social burden through the early detection and prevention of influenza epidemics.

### Limitation of the study

Our study has limitations to consider when interpreting the results. First, electronic health record system is not widely used in Japan; moreover, most rural clinics do not have an electronic medical records system, and instead, use a traditional paper medical record system. This includes the clinic where our research was conducted. Thus, we could not analyse the following data: (1) guardian characteristics (eg, age, sex, and academic background); (2) influenza vaccination status (influenza vaccination in Japan is provided on an outpatient bases under a free access system; therefore, records of influenza vaccinations are not linked to the patients’ information stored at clinics); (3) information on patients who did not visit the clinic. Second, the sample size of our study was very small although all influenza patients of the target age who underwent RIDT were included in the analysis. The research was conducted in a rural village with a population of about 500 individuals, including about 30 children aged <12 years. We retrospectively extracted limited data that quantitatively evaluated the self-diagnosis of influenza by guardians to their children. Therefore, a prospective study with a larger sample size, including data on guardian characteristics and influenza vaccination status, should be conducted in the future. Finally, our study was undertaken in a rural area of Japan. The reliability of the medical information was high owing to the somewhat isolated nature of the rural area. However, results might vary under different conditions, such as different regions (eg, urban versus rural areas), different patient backgrounds, and the influenza epidemic situation.

## Conclusion

We investigated the accuracy of self-diagnosis of seasonal influenza by guardians to their children. The 80% cut-off point showed high specificity, whereas the 50% cut-off point exhibited high sensitivity. The absence of feeling severely sick, as estimated by guardians, might be a useful indicator for ruling out paediatric influenza. Physicians should ask the guardians of the patients to provide their explanatory models to facilitate better discussion of the condition and elicit more accurate clinical diagnosis.

## References

[ref1] Chartrand C , Leeflang MM , Minion J , Brewer T Pai M (2012) Accuracy of rapid influenza diagnostic tests: a meta-analysis. Annals of Internal Medicine 156, 500–511. 22371850 10.7326/0003-4819-156-7-201204030-00403

[ref2] Copeland DL , Basurto-Davila R , Chung W , Kurian A , Fishbein DB , Szymanowski P Meltzer MI (2013) Effectiveness of a school district closure for pandemic influenza A (H1N1) on acute respiratory illnesses in the community: a natural experiment. Clinical Infectious Diseases 56, 509–516. 23087391 10.1093/cid/cis890

[ref3] Infectious Disease Surveillance Center. Infectious Agents Surveillance Report (IASR) (2010) Surveillance Data Table 2010 week 10. Retrieved 30 November 2017 from http://idsc.nih.go.jp/idwr/douko/2010d/10douko.html

[ref4] Infectious Disease Surveillance Center. Infectious Agents Surveillance Report (IASR) (2016) Weekly report of influenza virus isolation/detection, 2009/10-2015/16. Retrieved 30 November 2017 from http://www.nih.go.jp/niid/images/iasr/rapid/inf3/2016_19w/in1_160828.gif

[ref5] Jutel A , Baker MG , Stanley J , Huang QS Bandaranayake D (2011) Self-diagnosis of influenza during a pandemic: a cross-sectional survey. British Medical Journal Open 1, e000234. 10.1136/bmjopen-2011-000234 PMC3191601 22021887

[ref6] Jutel A Banister E (2013) “I was pretty sure I had the flu”: qualitative description of confirmed-influenza symptoms. Social Science & Medicine 99, 49–55. 24355470 10.1016/j.socscimed.2013.10.011

[ref7] Kai J (1996a) Parents’ difficulties and information needs in coping with acute illness in preschool children: a qualitative study. British Medical Journal 313, 987–990. 8892421 10.1136/bmj.313.7063.987 PMC2352333

[ref8] Kai J (1996b) What worries parents when their preschool children are acutely ill, and why: a qualitative study. British Medical Journal 313, 983–986. 8892420 10.1136/bmj.313.7063.983 PMC2352339

[ref9] Kanda Y (2013) Investigation of the freely available easy-to-use software ‘EZR’ for medical statistics. Bone Marrow Transplant 48, 452–458. 23208313 10.1038/bmt.2012.244 PMC3590441

[ref10] Kleinman A , Eisenberg L Good B (1978) Culture, illness, and care: clinical lessons from anthropologic and cross-cultural research. Annals of Internal Medicine 88, 251–258. 626456 10.7326/0003-4819-88-2-251

[ref11] Klepser ME (2014) Socioeconomic impact of seasonal (epidemic) influenza and the role of over-the-counter medicines. Drugs 74, 1467–1479. 25150045 10.1007/s40265-014-0245-1 PMC4149741

[ref12] Lang F , Floyd MR Beine KL (2000) Clues to patients’ explanations and concerns about their illnesses: a call for active listening. Archives of Family Medicine 9, 222. 10728107 10.1001/archfami.9.3.222

[ref13] Mitamura K Sugaya N (2006) Diagnosis and treatment of influenza--clinical investigation on viral shedding in children with influenza. Uirusu 56, 109–116. 17038819 10.2222/jsv.56.109

[ref14] Robinson JD Heritage J (2006) Physicians’ opening questions and patients’ satisfaction. Patient Education and Counseling 60, 279–285. 16431070 10.1016/j.pec.2005.11.009

[ref15] Thompson WW , Shay DK , Weintraub E , Brammer L , Bridges CB , Cox NJ Fukuda K (2004) Influenza-associated hospitalizations in the United States. Journal of the American Medical Association 292, 1333–1340. 15367555 10.1001/jama.292.11.1333

[ref16] Uchida M , Kaneko M , Yamamoto H , Honda T Kawa S (2013) Effects of school closure during influenza A/H1N1 pandemic in 2009 in Japan. Nihon Eiseigaku Zasshi. Japanese Journal of Hygiene 68, 103–117. 23718972 10.1265/jjh.68.103

[ref17] Wasserman R , Inui T , Barriatua R , Carter W Lippincott P (1984) Pediatric clinicians’ support for parents makes a difference: an outcome-based analysis of clinician-parent interaction. Pediatrics 74, 1047–1053. 6504624

[ref18] Woodall J , Rowson K McDonald J (1958) Age and Asian influenza, 1957. British Medical Journal 2, 1316. 13596596 10.1136/bmj.2.5108.1316 PMC2027356

